# Supplementary material for: Nuclear Magnetic Resonance and Calorimetric Investigations of Extraction Mode on Flaxseed Gum Composition
Source: Polymers (Basel). 2020 Nov 11;12(11):2654. doi: 10.3390/polym12112654 (PMC7697610; doi:10.3390/polym12112654)
Supplement: Supplementary file 1 [file polymers-12-02654-s001.pdf]

Supplementary material

NMR and calorimetric investigations of extraction mode on flaxseed gum composition

Fang Dubois[1], Corentin Musa[1], Benoit Duponchel[2], Lucette Tidahy[1], Xavier Sécordel[3], Isabelle Mallard[1], François Delattre [1,*]

S1:

^{1}H NMR spectra of the mucilage samples obtained at room temperature, 40 and 70 °C after different extraction duration (1, 20 and 48 hours) by traditional magnetic stirring method.

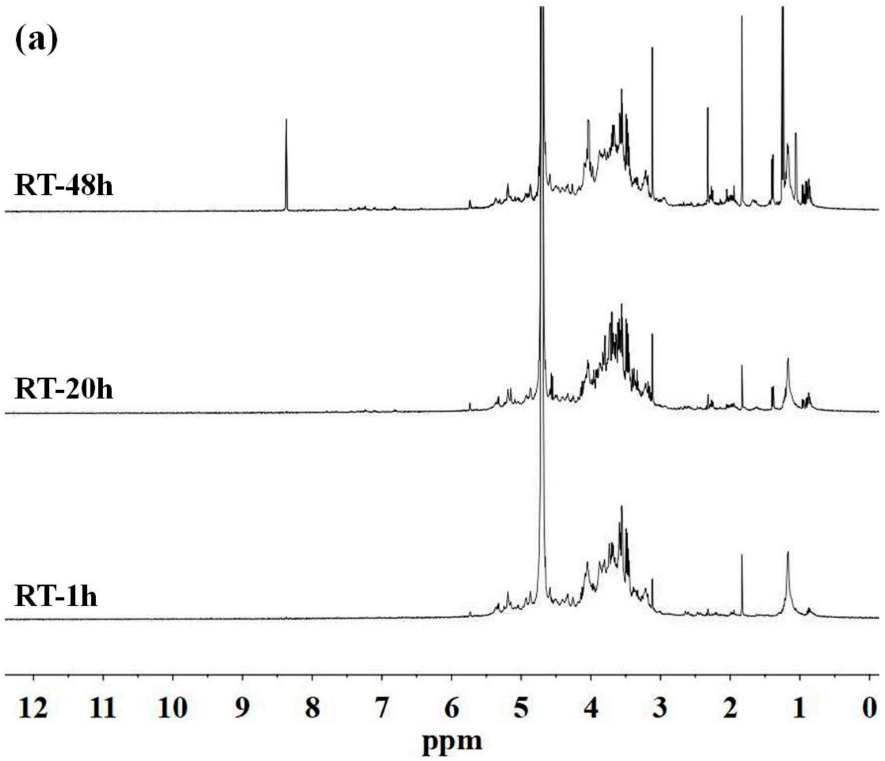

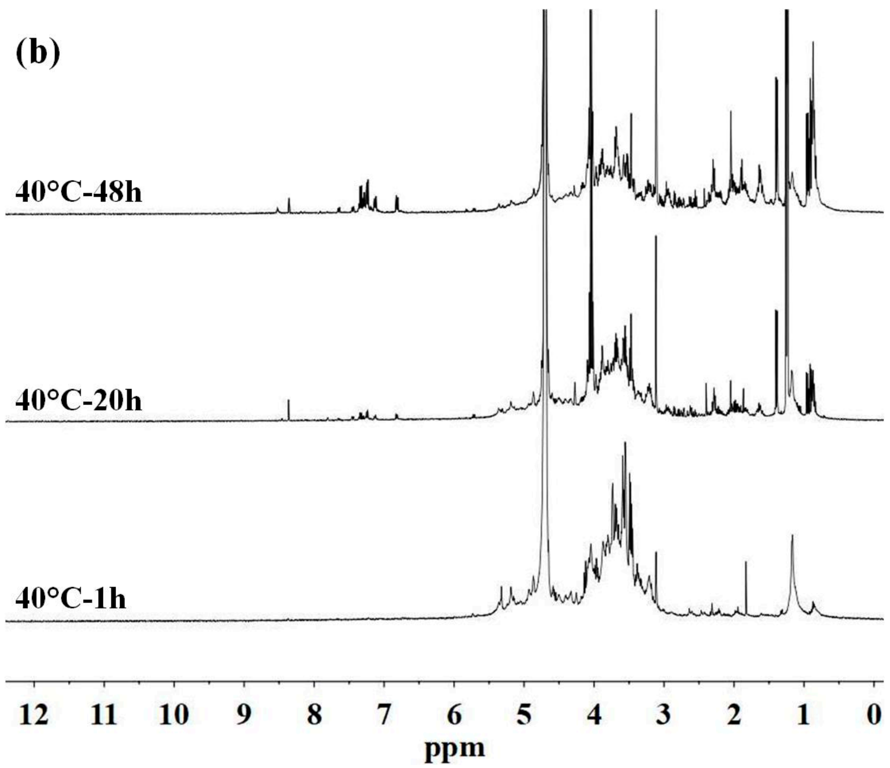
(b)
40°C-48h
40°C-20h
40°C-1h
12 11 10 9 8 7 6 5 4 3 2 1 0
ppm

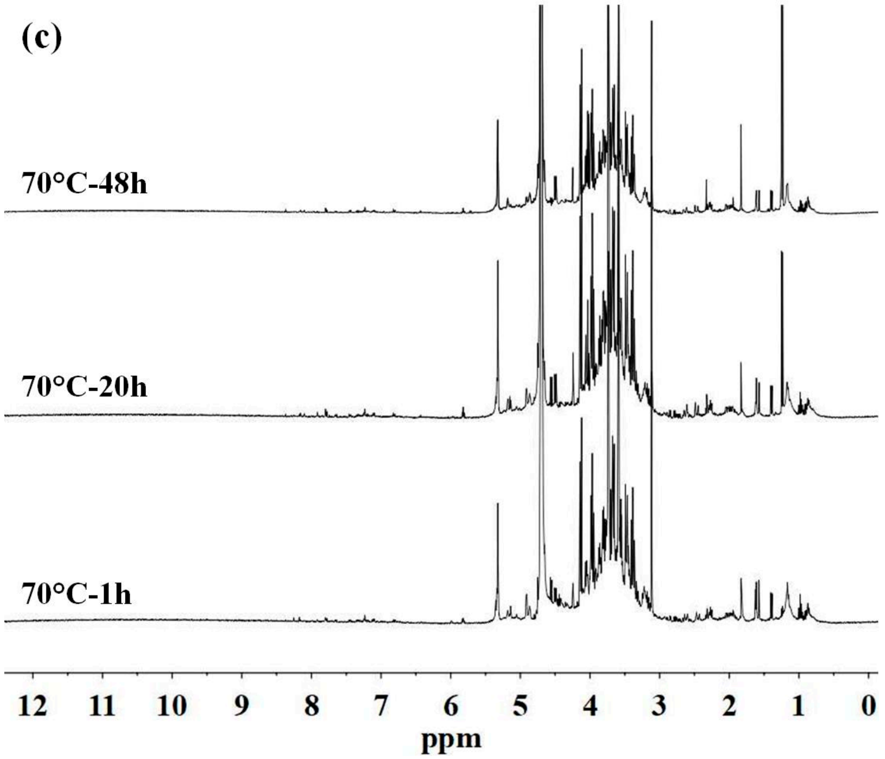
(c)
70°C-48h
70°C-20h
70°C-1h
12 11 10 9 8 7 6 5 4 3 2 1 0
ppm

S2:

^{13}C NMR spectra of the mucilage samples obtained at (a) room temperature, (b) 40 and (c) 70 °C after different extraction duration (1, 20 and 48 hours) by traditional magnetic stirring method.

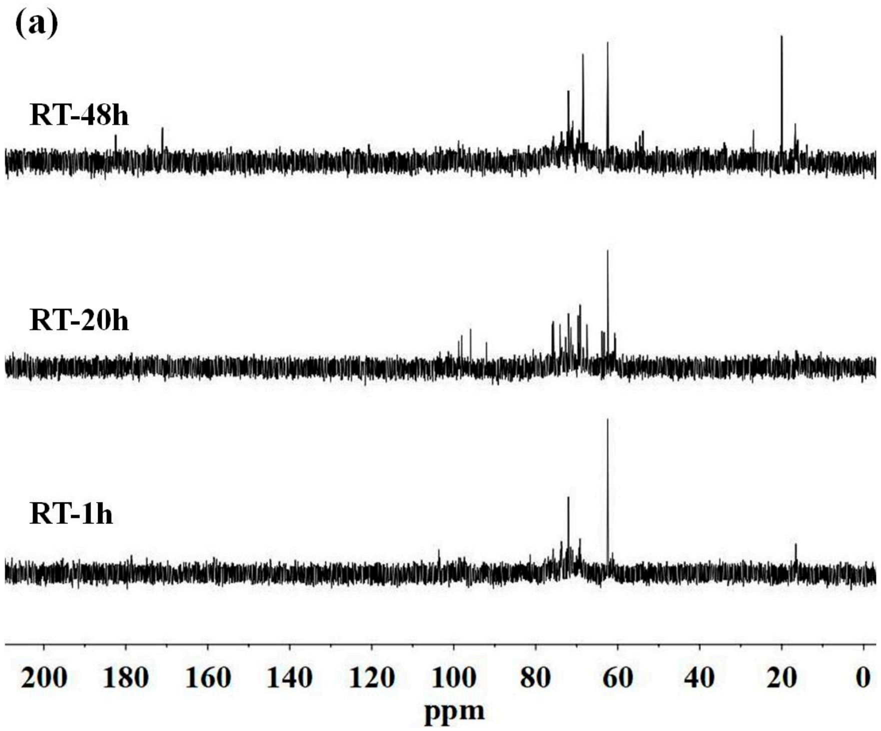

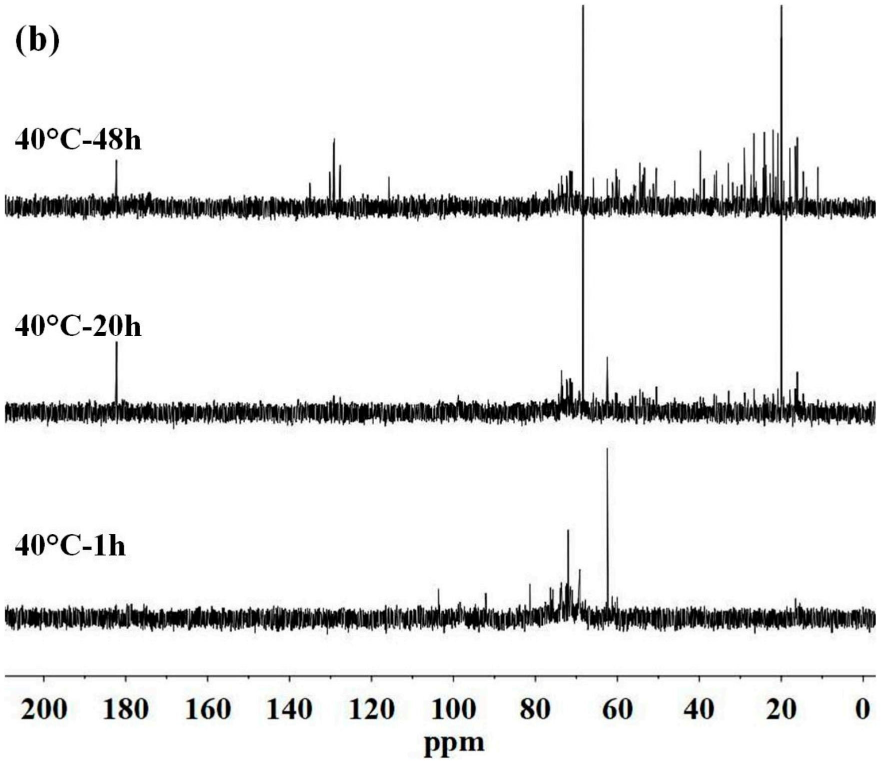

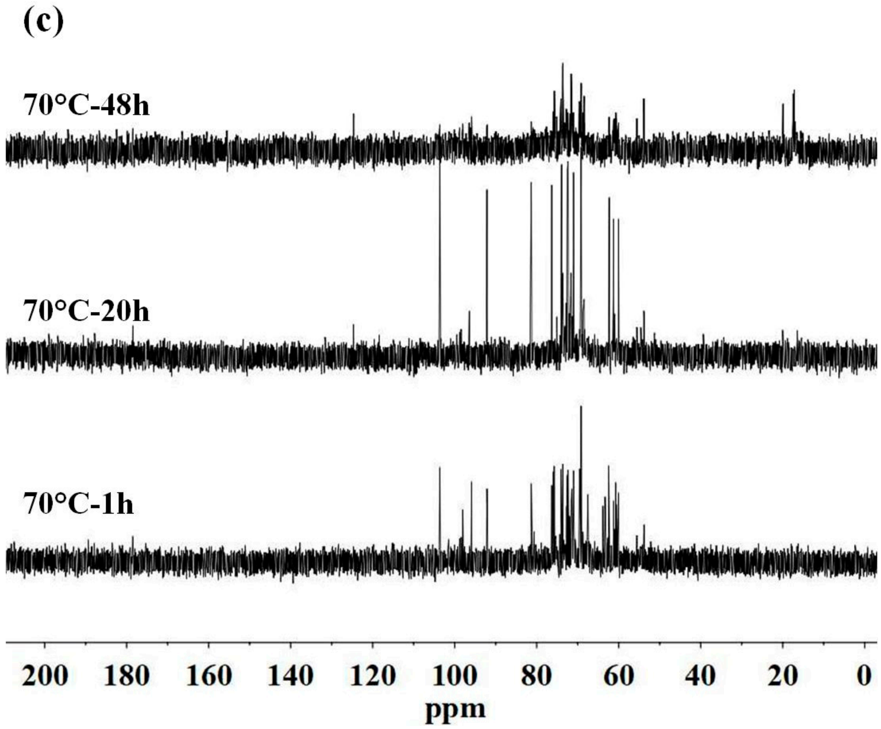
(c)
70°C-48h
70°C-20h
70°C-1h
200 180 160 140 120 100 80 60 40 20 0
ppm
